# Supplementary material for: The STRENGTH Study: A cluster randomised controlled trial of the effect of a behaviour change intervention added to cardiac rehabilitation on physical activity adherence
Source: PLoS One. 2026 Mar 24;21(3):e0345293. doi: 10.1371/journal.pone.0345293 (PMC13012500; doi:10.1371/journal.pone.0345293)
Supplement: S1 Table — (DOCX) [file pone.0345293.s001.docx]

S1 Table. Intra-class correlation co-efficients (ICCs) for key baseline physical and mental health measurements.

| Outcome | test | ICC | F | df1 | df2 | p | lower | upper |
| --- | --- | --- | --- | --- | --- | --- | --- | --- |
| Av. daily MVPA | ICC3k | 0.000 | 1.0 | 25 | 125 | 0.47 | -0.741 | 0.49 |
| Av. daily step counts | ICC3k | 0.322 | 1.5 | 25 | 125 | 0.085 | -0.181 | 0.66 |
| Age | ICC3k | 0.000 | 1.0 | 25 | 125 | 0.47 | -0.741 | 0.49 |
| BMI | ICC3k | 0.000 | 1.0 | 25 | 125 | 0.47 | -0.741 | 0.49 |
| EQ-VAS | ICC3k | 0.000 | 1.0 | 25 | 125 | 0.47 | -0.741 | 0.49 |
| WEMWBS | ICC3k | 0.000 | 1.0 | 25 | 125 | 0.47 | -0.741 | 0.49 |

*ICC3k removes mean differences between classes but are sensitive to interactions of participants by classes. It reflects the means of k participants and participants are seen as random effects.

Abbreviations: Intra-class correlation co-efficient (ICC), Moderate-Vigorous Physical Activity (MVPA), Body Mass Index (BMI), EuroQol Visual Analogue Scale (EQ-VAS), Warwick Edinburgh Mental Wellbeing Scale (WEMWBS).
