# Supplementary material for: The STRENGTH Study: A cluster randomised controlled trial of the effect of a behaviour change intervention added to cardiac rehabilitation on physical activity adherence
Source: PLoS One. 2026 Mar 24;21(3):e0345293. doi: 10.1371/journal.pone.0345293 (PMC13012500; doi:10.1371/journal.pone.0345293)
Supplement: S2 Table — (DOCX) [file pone.0345293.s002.docx]

S2 Table. Recruitment, data completeness and attrition rates across all timepoints.

| 1. Total no. participants recruited = 96 | | | | | | |
| --- | --- | --- | --- | --- | --- | --- |
|  | Questionnaires (N) | | | ActiGraph (that meet criteria of 4 days with at least 1 weekend day) | | |
| Timepoint | Total | Control | Intervention | Total | Control | Intervention |
| **Baseline** | 95 | 44 | 51 | 86 | 40 | 46 |
| **12 weeks** | 79 | 39 | 40 | 73 | 38 | 35 |
| **6 months** | 78 | 36 | 42 | 68 | 30 | 38 |
| **Baseline and 6 months** | 78 | 36 | 42 | 65 | 28 | 37 |
| **All timepoints** | 73 | 35 | 38 | 58 | 28 | 30 |
| B. Total no. participants dropped = 18 | | | | | | |
| Timepoint | Total | Control | Intervention |  |  |  |
| **After baseline** | 12 | 4 | 8 |  |  |  |
| **After 12 weeks** | 6 | 4 | 2 |  |  |  |

(A) Number of participants that were recruited into the study, with a further breakdown of numbers of complete and valid questionnaire and ActiGraph data at each timepoint; (B) Number of participants that dropped from the study cohort at each timepoint.
