# Supplementary material for: The STRENGTH Study: A cluster randomised controlled trial of the effect of a behaviour change intervention added to cardiac rehabilitation on physical activity adherence
Source: PLoS One. 2026 Mar 24;21(3):e0345293. doi: 10.1371/journal.pone.0345293 (PMC13012500; doi:10.1371/journal.pone.0345293)
Supplement: S3 Table — (DOCX) [file pone.0345293.s003.docx]

S3 Table. Adherence rates of physical activity (PA) tracking across the course of the study.

| Intervention contact timepoint | PA tracking adherence |
| --- | --- |
| Follow up 1 (weekly) | 98% |
| Follow up 2 (weekly) | 95% |
| Follow up 3 (weekly) | 95% |
| Follow up 4 (weekly) | 93% |
| Follow up 5 (weekly) | 89% |
| Follow up 6 (weekly) | 91% |
| Follow up 7 (monthly) | 66% |
| Follow up 8 (monthly) | 66% |
| Follow up 9 (monthly) | 73% |
