# Supplementary material for: The STRENGTH Study: A cluster randomised controlled trial of the effect of a behaviour change intervention added to cardiac rehabilitation on physical activity adherence
Source: PLoS One. 2026 Mar 24;21(3):e0345293. doi: 10.1371/journal.pone.0345293 (PMC13012500; doi:10.1371/journal.pone.0345293)
Supplement: S4 Table — (DOCX) [file pone.0345293.s004.docx]

S4 Table. Mean (± standard deviation) for daily moderate-vigorous physical activity (MVPA) and step counts at each timepoint.

| Timepoint | Control | Intervention |
| --- | --- | --- |
| **Baseline** |  |  |
| Daily MVPA (mins) | 40.93 (± 20.48) | 38.79 (± 26.37) |
| Daily step counts | 6341.52 (± 2422.65) | 6013.19 (± 3165.46) |
| **12 week** |  |  |
| Daily MVPA (mins) | 38.12 (± 21.36) | 39.72 (± 27.83) |
| Daily step counts | 6093.52 (± 2356.93) | 6313.19 (± 3368.52) |
| **6 month** |  |  |
| Daily MVPA (mins) | 39.98 (± 21.98) | 34.69 (± 23.87) |
| Daily step counts | 6386.81 (± 2409.96) | 6103.79 (± 3293.07) |
