# Supplementary material for: The STRENGTH Study: A cluster randomised controlled trial of the effect of a behaviour change intervention added to cardiac rehabilitation on physical activity adherence
Source: PLoS One. 2026 Mar 24;21(3):e0345293. doi: 10.1371/journal.pone.0345293 (PMC13012500; doi:10.1371/journal.pone.0345293)
Supplement: S5 Table — (DOCX) [file pone.0345293.s005.docx]

S5 Table. Median [and interquartile range] for Quality Adjusted Life Years at each timepoint.

| Timepoint | Control | Intervention |
| --- | --- | --- |
| **Baseline** | 0.88 [0.72 – 0.97] | 0.88 [0.78 – 0.97] |
| **12 week** | 0.88 [0.78 – 0.97] | 0.88 [0.87 – 0.97] |
| **6 month** | 0.88 [0.78 – 0.97] | 0.88 [0.79 – 0.97] |
