# Supplementary material for: The STRENGTH Study: A cluster randomised controlled trial of the effect of a behaviour change intervention added to cardiac rehabilitation on physical activity adherence
Source: PLoS One. 2026 Mar 24;21(3):e0345293. doi: 10.1371/journal.pone.0345293 (PMC13012500; doi:10.1371/journal.pone.0345293)
Supplement: S1 Fig — (DOCX) [file pone.0345293.s006.docx]

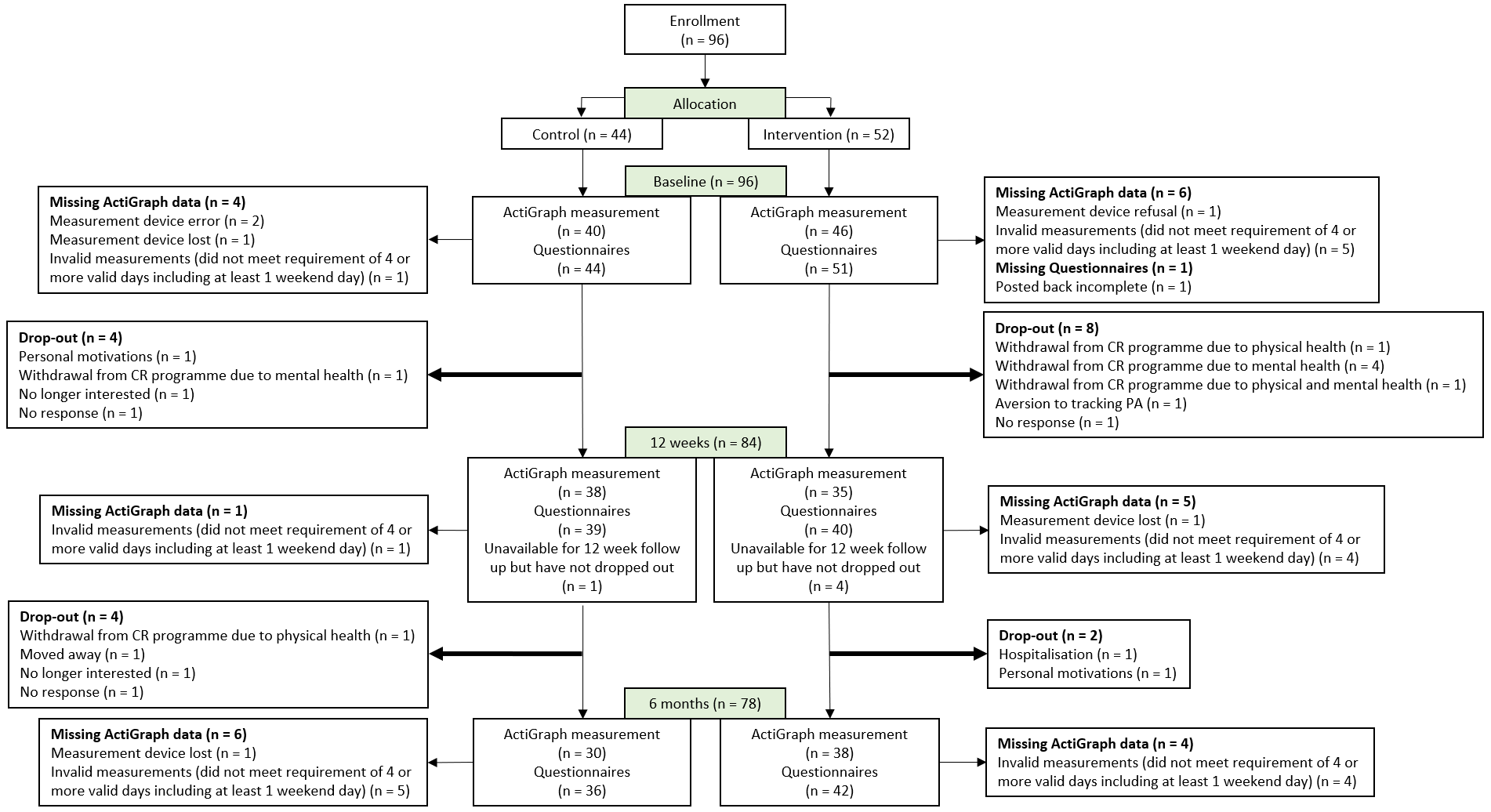


**S1 Fig. CONSORT participant flowchart of the STRENGTH cluster randomised controlled trial.**

In total, 96 patients were recruited for participation, random allocation to the intervention group or control group was conducted at the cardiac rehabilitation (CR) class level in a stepped-wedge design.
