# Supplementary material for: The STRENGTH Study: A cluster randomised controlled trial of the effect of a behaviour change intervention added to cardiac rehabilitation on physical activity adherence
Source: PLoS One. 2026 Mar 24;21(3):e0345293. doi: 10.1371/journal.pone.0345293 (PMC13012500; doi:10.1371/journal.pone.0345293)
Supplement: S2 Fig — (DOCX) [file pone.0345293.s007.docx]

**
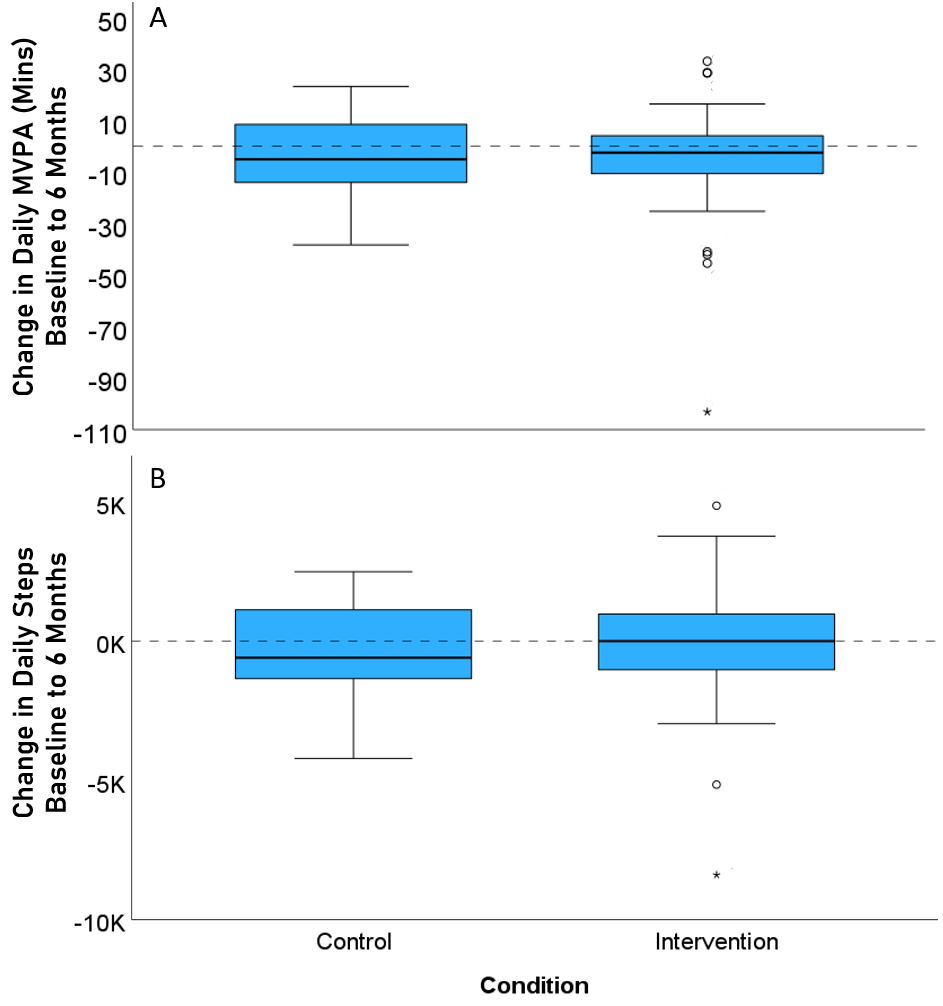
**

**S2 Fig. Change in daily physical activity for control versus intervention conditions from baseline to 6 months later.**

Boxplots represent median and interquartile range (IQR) for changes in daily: (A) moderate-vigorous physical activity (MVPA) in minutes, and (B) number of steps, achieved by participants in the control and intervention conditions. The whiskers extend to the smallest and largest values within 1.5 times the IQR. The additional data points are outliers: open circles (o) denote values 1.5-3 times beyond the IQR, stars () denote values >3 times beyond the IQR.
