# Supplementary material for: The STRENGTH Study: A cluster randomised controlled trial of the effect of a behaviour change intervention added to cardiac rehabilitation on physical activity adherence
Source: PLoS One. 2026 Mar 24;21(3):e0345293. doi: 10.1371/journal.pone.0345293 (PMC13012500; doi:10.1371/journal.pone.0345293)
Supplement: S3 Fig — (DOCX) [file pone.0345293.s008.docx]

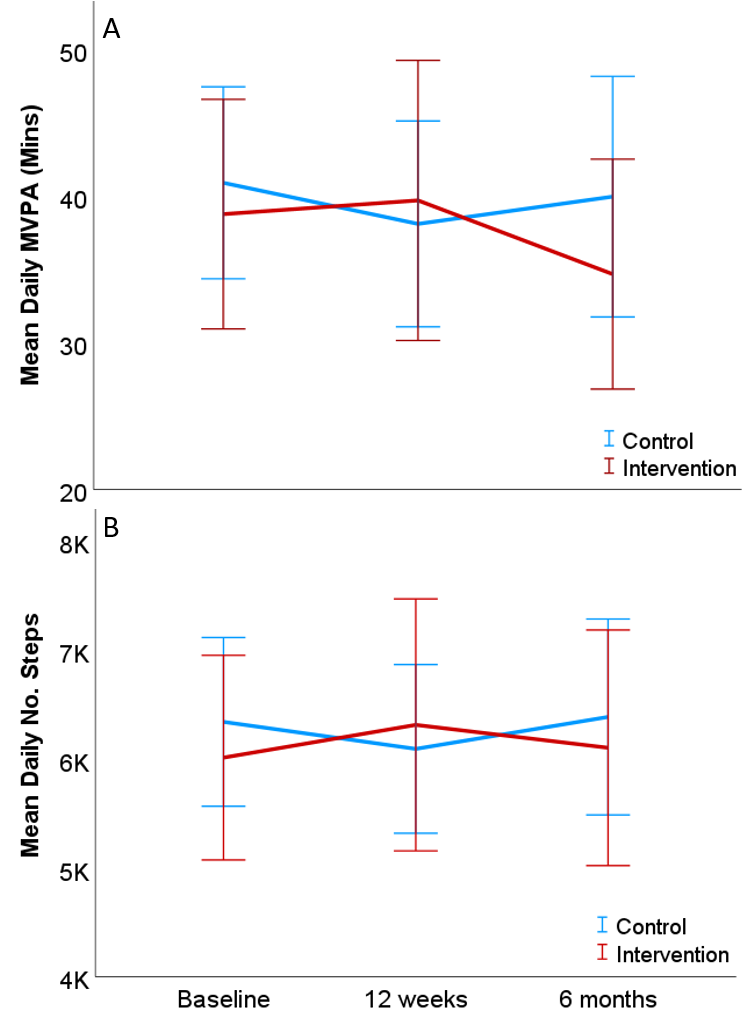


**S3 Fig. Mean daily physical activity for each condition, across all timepoints.**

Mean daily: (A) Moderate-vigorous physical activity (MVPA) in minutes, and (B) number of steps, at each timepoint (baseline, 12 weeks, and 6 months), for the control (blue) and intervention (red) conditions. Error bars represent 95% confidence intervals
