# Supplementary material for: The STRENGTH Study: A cluster randomised controlled trial of the effect of a behaviour change intervention added to cardiac rehabilitation on physical activity adherence
Source: PLoS One. 2026 Mar 24;21(3):e0345293. doi: 10.1371/journal.pone.0345293 (PMC13012500; doi:10.1371/journal.pone.0345293)
Supplement: S4 Fig — (DOCX) [file pone.0345293.s009.docx]

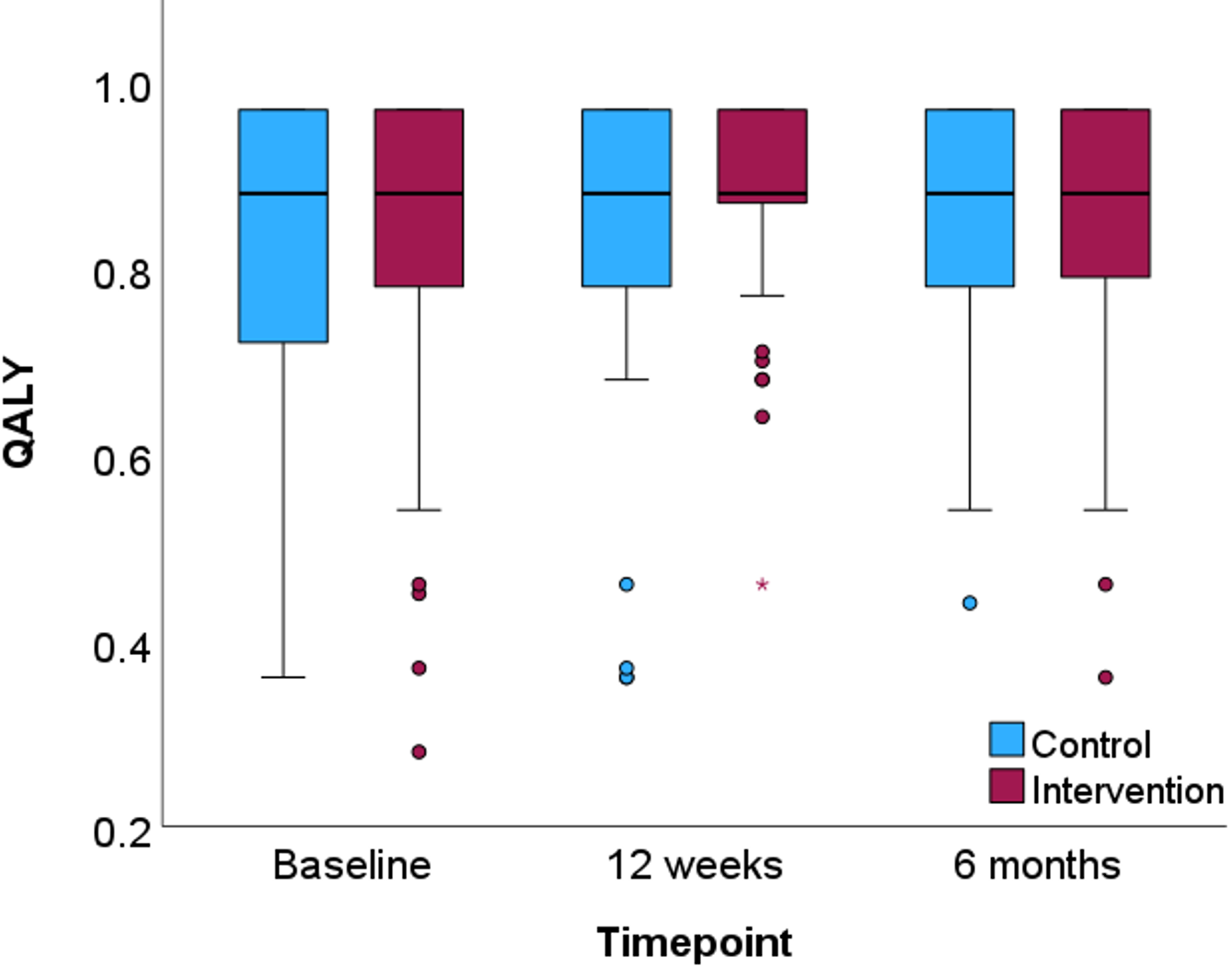


**S4 Fig.** **Boxplots represent median and IQR of QALYs at each timepoint**.

Control (blue) and intervention (red) conditions. The whiskers extend to the smallest and largest values within 1.5 times the IQR. The additional data points are outliers: circles (o) denote values 1.5-3 times beyond the IQR, stars () denote values >3 times beyond the IQR.
