# Supplementary material for: The STRENGTH Study: A cluster randomised controlled trial of the effect of a behaviour change intervention added to cardiac rehabilitation on physical activity adherence
Source: PLoS One. 2026 Mar 24;21(3):e0345293. doi: 10.1371/journal.pone.0345293 (PMC13012500; doi:10.1371/journal.pone.0345293)
Supplement: Attachment — Submitted filename: The STRENGTH Study - Response to Reviewers Comments.docx [file pone.0345293.s013.docx]

**Dr Nicole E. Blackburn**

Senior Lecturer

Centre for Health and Rehabilitation Technologies

Institute of Nursing and Health Research

School of Health Sciences

Derry~Londonderry

**Email:**  [ne.blackburn@ulster.ac.uk](mailto:ne.blackburn@ulster.ac.uk)

15 January 2026

Dear Editor,

We would like to thank all reviewers for their thorough and constructive feedback. We have carefully revised the manuscript in response to each point raised. Below, we provide a detailed response to all comments and indicate where changes have been made within the manuscript, which are clearly identified within the version with tracked changes.

| **Reviewer Comments** | **Response** |
| --- | --- |
| *Reviewer 1:*  *Thank you for giving me the opportunity to review this paper.* | *We thank Reviewer 1 for the detailed methodological critique, which has significantly strengthened the manuscript.* |
| Abstract It is misleading to state that 96 participants were randomised, as randomisation was conducted at the class (cluster) level. A more correct description would be that six cardiac rehabilitation (CR) classes were randomised, with participants following the allocation of their class. The study is therefore better classified as a cluster randomised trial rather than a true stepped-wedge trial, particularly since the stepped-wedge element is not actually utilised in the analyses. | The abstract has been revised to state that six CR classes were randomised as clusters and that participants followed the allocation of their class. We have removed wording suggesting individual randomisation. We have also revised the study description to clarify that although the study was originally planned as a stepped-wedge design, a formal stepped-wedge analytic approach was not applied, and only one step involved randomisation. This terminology is now used consistently throughout the manuscript. |
| Background Much of the cited literature is outdated (e.g. MacKay & Mensah 2004). The organisation and delivery of cardiac rehabilitation has changed substantially since then. Recent systematic reviews, especially the Cochrane review by Dibben 2021, as well as newer RCTs, should be included. References such as Dalal 2015 are insufficient as the main evidence base. The background section is generally weak and should be rewritten with up-to-date literature. | The background has been updated. We now refer to more recent systematic reviews, including Dibben et al. (2021, Cochrane Review), and incorporate current evidence from recent RCTs and observational studies. Older references have been removed unless historically relevant. The rationale for the study is now clearer and better supported by contemporary literature. |
| Methods The authors cite Hemming 2015, which is relevant, but more recent methodological references should be used (e.g. Chan, Leyrat & Eldridge 2024, Trials; Fan Li et al. 2022, World Neurosurgery). It is unclear whether all CR classes took place in different leisure centres or in the same centre. If the same centre was used, there is a considerable risk of contamination bias, as health professionals in control classes may have known the intervention. Table 1: Recruitment period 3 is not used and should be deleted. More generally, the rationale for choosing a stepped-wedge design is not clear, given that its features are not applied in the analyses. | We thank the reviewer for raising these important points. We have addressed each below:   1. **More recent methodology citations**   We have updated our methods section with contemporary references on cluster and stepped-wedge trial methodology, including Chan, Leyrat & Eldridge (2024) and Fan Li et al. (2022), which provide guidance on design, sample size, and analysis considerations for cluster-randomised trials and stepped implementation sequences.  **2. Whether classes shared centres**  Thank you for your comment. We have clarified the delivery setting in the revised manuscript. The same centre was not used. The study utilised five leisure centres across two counties in Northern Ireland, each of which delivered at least one CR class during the study period. Because several centres run multiple maintenance-stage CR classes annually, individual centres could host more than one study class and therefore could include both control and intervention participants at different points in recruitment. The standard exercise component of CR was delivered uniformly across all centres. The behaviour-change component, however, was delivered only to participants in the intervention arm, in small intervention groups and, where required due to scheduling or participant needs, individually. These sessions took place separately from the routine CR classes, which reduced, but could not entirely eliminate the possibility of contamination through informal participant interactions within the same centre.  **3. Table 1 includes unused period and rationale for stepped-wedge design**  Thank you for these helpful comments. We address each point below.  **Recruitment Period 3:** Recruitment Period 3 was used during the study. Participants were enrolled during this phase, and the period formed part of the planned implementation schedule. We have clarified this in the manuscript to ensure that the role of Recruitment Period 3 is explicit. For transparency, we have retained Recruitment Period 3 in Table 1 and have updated the accompanying text to state that all three recruitment periods contributed to participant enrolment.  **Rationale for the stepped-wedge design:** We agree that the analyses ultimately did not use stepped-wedge modelling. As now fully detailed in the revised Methods section, the study was originally designed as a stepped-wedge cluster randomised trial. This design was selected because it aligned well with the pragmatic service-delivery context, allowed staging of implementation across centres, and supported the planned gradual roll-out of the behaviour-change intervention. However, due to operational constraints, including disruptions to centre schedules and capacity during the COVID-19 period, the implementation sequence could not be executed in the uniform waves required for formal stepped-wedge analysis. As a result, the analytic approach was adapted prior to analysis. The implemented study therefore aligns more closely with a cluster randomised trial with a stepped implementation sequence, rather than a full stepped-wedge CRT. While a formal stepped-wedge analytic approach was not applied, the staged roll-out across centres still enabled examination of implementation and intervention effects across clusters within the pragmatic constraints of service delivery. |
| Statistical analyses  The sample size calculation is insufficiently described and cannot be reproduced. It is unclear how the ICC was estimated. A statistician should review this section. The study initially planned for 15 clusters, but only 6 CR classes were included (with 4 implemented as planned). This is a major limitation, as the required sample size was not achieved, severely reducing power and validity. See Chan, Leyrat & Eldridge (2024) for proper handling of cluster stepped-wedge sample size and analyses. | We thank the reviewer for these important methodological points. We have clarified the sample-size justification and added further explanation to enhance transparency.  **Sample size reproducibility:**  The target sample (n=100) was based on effect-size estimates from our previous work (Cupples et al., 2013), which reported a between-group difference of approximately 2,500 steps/day (SD 3,500). This effect size, combined with 80% power and α = 0.05, produced the required sample size when incorporating a 15% attrition allowance.  **ICC rationale:**  The ICC of 0.01 used in the original power calculation was drawn from typical ICC ranges reported in similar community-based physical-activity and rehabilitation studies. Once data collection was completed, we calculated observed ICCs across clusters (CR classes) for key outcomes using R. These were uniformly very low (as shown in Supplementary Table 1), supporting the small ICC assumption used in the a priori calculation.  **Number of clusters:**  We agree that stepped-wedge designs ideally require a larger number of clusters to maximise efficiency. However, this study was embedded within real-world cardiac rehabilitation delivery across Northern Ireland, where only five leisure centres (hosting 15 total study classes) were available. The analytic plan was therefore adapted to align with the pragmatic constraints of the service environment. As we now state in the manuscript, the final analytic approach corresponded to a cluster trial with staggered implementation rather than a full stepped-wedge approach. We have added text acknowledging the reduced number of clusters as a limitation.  **Statistician input:**  The statistical analysis plan, including power calculations, ICC estimation, and selection of non-parametric and ANOVA approaches, was developed in consultation with an experienced statistician. We have added a statement to the Methods to make this more explicit. |
| Results The results section should start by clearly stating the number of classes and participants in each class. There is inconsistency between the text (line 229: six classes) and Table 1 (4+2). This needs clarification. Participants appear healthier, better educated, and more physically active than the typical CR population. This selection bias substantially limits generalisability. A dropout analysis is missing: baseline values for those not included or who dropped out should be presented (age, sex, education, living situation, comorbidity, etc.). It is also unclear who received further CR after the 12 weeks (lines 300–306). At minimum, numbers and extent of CR participation should be reported. Without these data, it is impossible to evaluate the independent effect of the intervention. | **Number of CR classes and centres**  We appreciate the opportunity to clarify the structure of the CR delivery sites. Across Northern Ireland, five leisure centres participated in the study. Each centre hosted multiple CR classes, resulting in 15 study classes in total. Because recruitment occurred continuously as patients enrolled in routine CR, some classes contained a mix of control and intervention participants, but the behaviour change component was delivered only to those randomised to the intervention condition, typically in small groups and, where necessary, individually.  **Potential for selection bias**  We acknowledge that the pragmatic, service-embedded design creates potential for selection bias. However, several steps were taken to minimise this risk:  • All eligible patients entering participating CR programmes during recruitment periods were approached consecutively, reducing the likelihood of systematic differences in who was invited or enrolled. • Baseline analyses confirmed there were no significant differences between intervention and control participants on demographic or clinical variables. • Allocation followed the predefined staggered implementation schedule, not participant choice or staff selection. We have added this clarification to the methods and have acknowledged selection bias as a potential limitation in the Discussion.  **Missing dropout analysis**  We agree that attrition warrants clearer reporting. The study experienced some loss to follow-up, largely due to illness, lack of valid data or disengagement from routine CR. We did not perform a formal dropout analysis because of the limited sample size, which increases the risk of spurious findings. We have now clearly stated this rationale in the Methods.  **Extended CR participation**  We recognise that the role of extended CR participation could be more clearly explained. All participants were enrolled in standard Phase IV CR delivered within leisure centres. The behaviour change intervention was added to, rather than replacing, their routine CR programme. Attendance at extended CR sessions was recorded but was not used as a covariate because (1) attendance patterns were highly variable, and (2) observed ICCs indicated that class-level clustering, including class attendance, did not meaningfully contribute to variance in outcomes. We have now clarified this in the Methods and included a brief statement in the Discussion noting that varying levels of CR participation may have influenced behaviour change trajectories. |
| Discussion & Limitations The discussion mentions high baseline activity levels and extended CR access as possible explanations, but these should have been addressed as design weaknesses already in the methods. The most important limitations are: 1. Misclassification as a stepped-wedge trial. 2. Insufficient statistical power due to too few clusters. 3. Risk of contamination if intervention and control classes were delivered at the same site. 4. Strong selection bias (participants not representative of the CR population). 5. Missing dropout analysis. 6. Lack of clarity on extended CR after the intervention. 7. Outdated background literature. | We have expanded the limitations and methods sections to clarify the final study design, acknowledge reduced statistical power, potential selection bias and contamination, dropout considerations, and variability in CR exposure. The background has also been updated to reflect current evidence. |
| Conclusion The study suffers from serious methodological flaws: it is underpowered, the participants are not representative, the design is misclassified, and the analyses do not utilise stepped-wedge methodology. In addition, the background is outdated, and essential analyses such as dropout characteristics and extended CR participation are missing. For these reasons, I find the manuscript unsuitable for publication in its current form. | The conclusion has been rewritten to reflect the exploratory nature of the findings given the methodological constraints, reduced power, and the modified design structure. |
| *Reviewer 2: Thank you for the opportunity to review this manuscript, describing the results of a stepped-wedge RCT of a behaviour change intervention added to maintenance cardiac rehabilitation compared to standard maintenance CR alone. The manuscript is written well, and although no effects were observed, the findings remain important. However, several key details about the intervention and control conditions are missing, which impacts the reliability and replicability of the study. The authors may wish to consider the following points:* | *We thank Reviewer 2 for their positive appraisal and helpful requests for clarification.* |
| General: Consistency of terminology. Throughout the manuscript, the intervention is described in a variety of ways, (e.g. “behaviour change intervention” in the title, “self-management intervention” in the abstract, and “multi-component intervention” in the background. A consistent term should be used throughout. | We now refer consistently to a behavioural change intervention throughout the manuscript. |
| Background: It could be made clearer that cardiac rehabilitation aims to increase both physical fitness and physical activity levels through its various components. A more detailed discussion of why an increase in PA is harder to achieve/sustain would strengthen the rationale. This could be linked to the potential value of interventions that incorporate behaviour change techniques. | We now make explicit that CR aims to improve both physical fitness and physical activity. We expanded the discussion of difficulties maintaining PA post-CR and the rationale for behaviour change support. |
| Methods: Please provide more detail of the prior CR (phase III) received by participants? For example, how long was the gap (if any) between completion of CR and the start of the maintenance intervention? Please provide more details of what the standard CR maintenance programme involved. How many sessions per participants expected to attend per week? How long did the sessions last? Were they delivered in-person? Did the sessions involve participation in an exercise programme? If so, what was the exercise prescription? As the intervention is described as a behaviour change intervention, I would expect to see some description of the behaviour change techniques used, and the mechanisms by which they were expected to work. The duration and content of the behaviour change intervention is not clear. What took place for the first six weeks before the pedometers were handed out? How many group discussion sessions were there? How long were these sessions? What was the typical number of participants involved in each class? Was there any patient and public involvement in the intervention development or other aspects of the study? | **Prior completion of Phase III CR and time gap before study enrolment**  We agree that additional detail regarding participants’ prior CR is important for interpreting the findings. All participants had previously completed standard Phase III cardiac rehabilitation. However, due to disruptions caused by COVID-19 service pauses and delays, the interval between completion of Phase III and enrolment into the study varied considerably. While some participants entered the study approximately 3 months post–cardiac event, others had completed Phase III up to 2 years earlier. We have now added this information to the Methods and acknowledged in the Discussion that this variability may have influenced readiness for further behaviour change.  **Standard CR maintenance programme (Phase IV) content and structure** The study was embedded within routine Phase IV community-based CR delivered in leisure centres. The typical programme included: • One supervised classes per week, lasting approximately 60 minutes. • Sessions delivered in-person by leisure-centre exercise professionals trained in CR delivery. • A structured exercise component comprising warm-up, aerobic activity (e.g., treadmill, cycling, step-based routines), resistance exercises, and cool-down. • Exercise intensity was guided by national CR recommendations (moderate intensity; Borg RPE 11–14), with individual tailoring based on functional capacity and clinical history.  We have added this detail to the manuscript to more clearly describe the standard care received by both groups.  **Behaviour change intervention: content, duration, and theoretical basis**  We have clarified the behaviour change intervention in the Methods. The 12-week intervention incorporated established behaviour change techniques (BCTs) including goal-setting, self-monitoring, problem-solving, action planning, social support, and feedback. During the first six weeks, participants engaged in weekly reviews (in-person or by phone) to set realistic step-count goals, identify barriers, and plan strategies, before receiving pedometers to support self-monitoring. Group discussions led by the researcher focused on PA benefits, local activity opportunities, and overcoming barriers, with sessions typically lasting 20–30 minutes and including 2–6 participants per discussion. After the first six weeks, participants received three further monthly check-ins to support ongoing behaviour regulation. These details have now been explicitly included in the Methods to clarify content, duration, and theoretical mechanisms.  **Patient and Public Involvement (PPI)**  Yes, PPI informed the study. Patient representatives from local CR user groups contributed to the design of study materials (e.g., recruitment materials, clarity of instructions) and provided feedback on acceptability of the intervention components. Their input helped ensure that the intervention and study processes were acceptable, feasible, and aligned with patient priorities. This has now been added to the manuscript. |
| Results/Discussion: Did the participants who dropped out of the study differ from those who remained? If the information is available, it would be useful to know what CHD diagnoses the participants had. Please specify what costs were included in the calculations for health service use? Was any adjustment made for multiplicity, given that there were two primary outcomes? If not, this should be acknowledged in the limitations. How much might the varying levels of adherence to the intervention have impacted the results? E.g., 55% goal setting. | **Differences between participants who remained and those who dropped out** We examined available baseline characteristics for participants who withdrew compared with those who completed follow-up. There were no statistically significant differences in key demographic or clinical variables (age, sex, BMI, baseline PA measures) between completers and non-completers. However, because the sample size was modest and reasons for dropout were not consistently recorded, we recognise that unmeasured factors may have contributed.  **CHD diagnoses of included participants** Detailed CHD diagnoses of participants were not collected. All participants had completed core CR and were clinically assessed and risk stratified according to BACPR guidelines.  **Costs included in health-service utilisation calculations** Our cost analysis included the standard categories recommended in UK CR economic evaluations. These categories have been explicitly added to the Methods. No indirect costs (e.g., productivity loss, informal care) were included.  **Adjustment for multiplicity of primary outcomes** We agree that having two primary PA outcomes (daily MVPA and daily step counts) introduces a risk of Type I error. No formal adjustment for multiplicity (e.g., Bonferroni correction) was applied, due to the exploratory nature of the analysis and the limited sample size. We have now explicitly acknowledged this in the limitations section and clarified the rationale for not adjusting.  **Impact of varying adherence to the intervention** Adherence to the behaviour change elements varied, in part due to COVID-related disruptions and fluctuating class attendance. Reduced exposure to behaviour change components likely diluted the intervention effect, given that goal-setting, action planning, and self-monitoring are core mechanisms through which PA behaviour change is achieved. We have added commentary in the Discussion highlighting that inconsistent adherence, particularly to early behaviour change tasks, may have limited the intervention’s effectiveness and should be addressed in future implementation. |
| *Reviewer 3: This is evaluating the effectiveness of a multi-component intervention incorporating current evidence from previous research and applying it to a maintenance stage CR programme to promote sustained changes in PA following completion.* | *We thank Reviewer 3 for these constructive comments.* |
| The sample size is based on one of the outcomes - yet the primary outcome section states they are potentiall two, i.e steps per day and time spent in moderate vigorous PA (MVPA) per day. Either the sample size needs to reflect the both outcomes, or clarity whether outcome number 2 is a secondary outcome? | We clarified that two primary outcomes were defined: daily step counts and daily MVPA. The sample size calculation was based on daily step counts, considered the most clinically relevant outcome. No formal adjustment for multiplicity was applied due to the exploratory nature of the study and the limited sample size; this has now been acknowledged in the Discussion as a limitation. |
| Under study design - suggest to start the sentence with the stating what the study design, e.g "This is a stepped-wedged cluster randomised trial". details regarding the reporting guidelines - this should actually be under statistical analyses as you are stating how the study will be report. | The study design section has now been updated and we explain the deviation from the original stepped-wedge plan. |
| In terms of recruitment - line 59, there is mention of 6 CR classes across 2 counties, with table 1 showing the allocation sequence, across these counties, can you have more than one CR class within each county? Can the distinct randomisation unit be made clear? Ah I see its mentioned in line 229 - this should probably be in the methods section. | We now clearly state that randomisation occurred at the class (cluster) level. |
| Under statistical analysis - it would be good to define population for analyses, since line 143 suggest, a criteria needed to be met. | We have clarified the analysis population. Participants were included in primary outcome analyses only if they met pre-specified accelerometer wear-time criteria (≥4 valid days, including ≥1 weekend day, ≥10 hours/day), ensuring that included data reflect habitual physical activity. |
| Can an explanation be given to explain the delay in recruitment, i.e line 60 says planned recruitment period was Mar 2022, but actual date reported in results section says Mar 2023 (line 203). | We have added a brief explanation for the delay from planned to actual recruitment, citing service pressures and post-pandemic restructuring. |

We sincerely thank all reviewers for their insightful and constructive feedback. In response, the manuscript has undergone substantial revision to:

- Address methodological concerns, including sample size, ICC, and cluster structure;
- Clarify the study design as a cluster randomised trial with a stepped implementation sequence, and explain why a formal stepped-wedge analytic approach was not applied;
- Enhance transparency regarding recruitment, class delivery, prior CR participation, intervention content, and potential sources of bias;
- Strengthen reporting of outcomes, dropout analyses, adherence, and cost-effectiveness methods; and
- Explicitly acknowledge study limitations, including variability in participant adherence, potential contamination, and reduced statistical power due to the limited number of clusters.

These revisions ensure that the manuscript more accurately reflects the study’s real-world context, scope, and constraints, while providing a clear and transparent account of the intervention’s implementation and findings.

If you have any queries, please feel free to get in touch.

Yours sincerely,

Dr Nicole Blackburn
